# Seroprevalence of antibodies against *Borrelia burgdorferi* sensu lato in healthy blood donors in Romania: an update

**DOI:** 10.1186/s13071-021-05099-1

**Published:** 2021-12-04

**Authors:** Zsuzsa Kalmár, Violeta Briciu, Mircea Coroian, Mirela Flonta, Amanda-Lelia Rădulescu, Adriana Topan, Andrei Daniel Mihalca, Mihaela Lupșe

**Affiliations:** 1 “Iuliu Hațieganu” University of Medicine and Pharmacy Cluj-Napoca, Cluj-Napoca, Romania; 2 Hospital for Infectious Diseases, Cluj-Napoca, Romania; 3 University of Agricultural Sciences and Veterinary Medicine Cluj-Napoca, Cluj-Napoca, Romania

**Keywords:** Lyme disease, Seroprevalence, *Borrelia burgdorferi*, Antibodies, *Ixodes ricinus*

## Abstract

**Background:**

The *Borrelia burgdorferi* sensu lato (s.l.) genogroup is the causative agent responsible for Lyme borreliosis, a common tick-borne infectious disease in some temperate regions of the Northern Hemisphere. In humans, the clinical manifestations of Lyme borreliosis vary from dermatological infection to severe systemic manifestations. In Romania, data on the seroprevalence of Lyme borreliosis and associated risk factors are scarce and outdated, as the only seroprevalence study with a large dataset was published more than 20 years ago. Therefore, the aim of the present study was to evaluate the seroprevalence for *Borrelia burgdorferi* s.l. in healthy blood donors from six Romanian counties and identify the associated risk factors.

**Methods:**

The study was conducted among 1200 healthy blood donors aged between 18 and 65 years during November 2019 and September 2020 from six counties in the northwestern and central parts of Romania. A two-tiered testing strategy was applied. Positive and equivocal immunoenzymatic test results for IgG and IgM antibodies were further confirmed by Western blot.

**Results:**

Serum samples from 20% of the blood donors had positive or equivocal IgG and IgM ELISA index values. In total, 2.3% of the serum samples for IgG and 1.8% for IgM were positive by Western blot. The seroprevalence for both antibodies varied between 1.5% (Satu-Mare) and 6.5% (Bistrița-Năsăud) in the six counties investigated. The highest seroprevalence was observed in men (4.7%), in blood donors performing their professional activities outdoors (4.2%), and in those aged  ≥ 56 years (8%).

**Conclusions:**

These findings confirm the presence of specific IgG and IgM antibodies to *B. burgdorferi* s.l. among healthy blood donors from Romania. Furthermore, potential risk factors, such as gender, age, and behavior, associated with the presence of positive *B. burgdorferi* s.l. antibodies among healthy blood donors were identified.

**Graphical Abstract:**

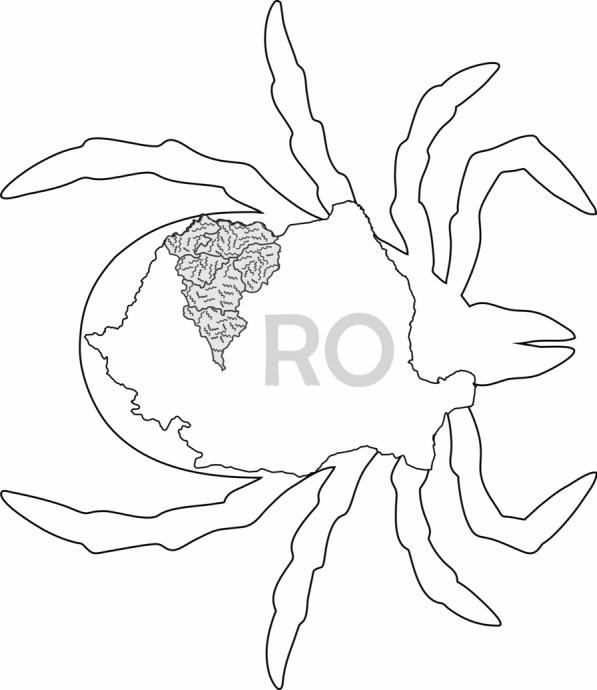

## Background

In Europe, due to the high climatic and habitat heterogeneity favorable for tick vectors, Lyme borreliosis (LB) and other vector-borne diseases are increasing. The *Borrelia burgdorferi* sensu lato (s.l.) genogroup is the causative agent responsible for the tick-borne zoonosis LB [1]. The life cycle of LB-causing spirochetes involves small mammals and birds, vectorized by ticks. The main vector in Europe is represented by *Ixodes ricinus*, also widely distributed in Romania [2].

Lyme borreliosis is currently the most commonly reported vector-borne disease in Europe and North America and is prevalent in temperate areas of Asia [3]. In humans, the clinical manifestations of LB vary and are more or less specific, causing dermatological, rheumatological, neurological, ophthalmological, and cardiac symptoms [3]. In Romania, over the past 10 years, the incidence of LB has varied between 1.2 and 4.2 cases per 100 000 individuals annually [4], although the real number of diagnosed cases may be widely underreported [5]. The clinical case definition in Romania for the diagnosis of early cutaneous LB is different from most European countries’ guidelines [6], as it recommends early sampling for serological tests in patients with suspected erythema migrans [5]. Diagnostic difficulties, under- and overreporting, and different laboratory methods used are important issues for LB diagnosis and surveillance [7]. Thus, case definitions may vary according to different public health authorities, and consensus on a standardized case definition between EU countries is needed [8].

In Europe, a two-tiered methodology, using indirect diagnostic tools such as serological assays, is recommended in national and international guidelines for the serodiagnosis of LB [9]. The first-step assay is usually performed using an enzyme-linked immunosorbent assay (ELISA) or indirect immunofluorescence assay, and in the second step an immunoblot [Western blot, (WB), line-blot, or dot-blot] is used to confirm or rule out positivity. To avoid unnecessary antibiotic therapy, in addition to serological screening, clinical and epidemiological data are recommended and are essential for the diagnosis [9]. There are several risk factors for developing LB, such as the density of the tick population, the rate of tick infection with *Borrelia* spp., and the duration of tick attachment to humans [10]. Outdoor activity for work or leisure is also an important risk factor for developing LB. Monitoring LB in a defined population group who engage in outdoor activities (forestry workers, farmers, soldiers) in highly endemic geographical regions is of particular importance in assessing fluctuations in human infection risk [11].

Despite the deficient reporting system, studies on the occurrence of tick-borne pathogens have increased in recent years in Romania. So far, the largest cross-sectional observational study regarding *B. burgdorferi* s.l. seroprevalence in healthy blood donors and forestry workers in our country was conducted in 1999 by Hristea et al. [12]. Briciu et al. [13] also assessed the presence of *B*. *burgdorferi* s.l. antibodies in patients who reported tick bites in Cluj County during 2010, and investigated the presence of *Borrelia* spp. DNA in the detached ticks. The average local prevalence of *Borrelia* spp. in questing *I. ricinus* in Romania varies widely according to the geographical region [14–17], and prevalence varying between 3.4 and 14.2% in *I. ricinus* collected from humans was reported in Cluj and Sibiu counties [18]. Aside from several studies on the presence and distribution of tick-borne pathogens in questing [19, 20] and engorged ticks collected from humans [21] and wild hosts [17, 22–28], and serological surveys in dogs and horses [29–31], reports on the incidence of tick-borne bacterial diseases in humans still remain limited. As the only seroprevalence study with a large dataset was published more than 20 years ago, and because data concerning the prevalence of tick-borne diseases in humans from Romania are outdated and scarce, further up-to-date research could be beneficial to the Romanian population for awareness campaigns.

The aim of this study was to assess the seroprevalence of immunoglobulin G (IgG) and IgM antibodies against *B. burgdorferi* s.l. in healthy blood donors from six counties in Romania and to identify the associated risk factors. Although seroprevalence studies are based mainly on IgG analyses, because positive IgM results may persist for years after acute LB [9, 32–35] and serological profiles after erythema migrans have shown a persistent IgM profile and lack of seroconversion of IgG antibodies [35], we also evaluated the IgM seroprevalence in the study group.

## Methods

### Sample collection

Human blood was collected by medical personnel from regional blood transfusion centers from healthy blood donors in six counties (Alba, Bistrița-Năsăud, Cluj, Maramureș, Sălaj, Satu-Mare) from the northwestern and central parts of Romania. In Romania, the National Institute of Public Health is organized in six regional centers; the Regional Institute of Health Cluj coordinates the public health activity of the six counties included in our study. Blood collection was performed between November 2019 and February 2020 and between August and September 2020. A questionnaire was filled in by each patient regarding residence, age, gender, occupation, and urban/rural environment. Blood donors were categorized by age into young (age  ≥ 18 ≤ 35 years), middle-aged (age  ≥ 36 ≤ 55 years) and old (age  ≥ 56 years) adults. The blood samples were centrifuged at 0.8 relative centrifugal force (rcf) for 10 min and stored at –80 °C until further serological analysis. The following variables were collected: age, gender, residence, occupation, education.

#### Ethics statement

Each patient was informed of the aims and the protocol of the study. All blood samples were obtained following informed consent. The study was approved by the National Institute of Hematology and Blood Transfusion, Romania (Registration Number: 2589/c/24.oct.2019).

#### Serological analysis

According to national and international guidelines, a two-stage serodiagnostic (screening and confirmation) testing strategy was applied in order to assess the seroprevalence of *B. burgdorferi* s.l. in human sera. First, blood serum samples were screened by ELISA using the *recom*Line Borrelia IgG/IgM (Mikrogen Diagnostik, Germany) kit to determine the presence of IgG and IgM *B. burgdorferi* s.l. antibodies. All ELISA-positive and equivocal serum samples were analyzed by WB using the *recom*Well Borrelia IgG/IgM (Mikrogen Diagnostik, Germany) kit for confirmatory testing. WB was performed and interpreted using the *recom*Scan automated scanner and reader and the test strip analysis software (Mikrogen Diagnostik, Germany). The sum of the points attributed to each antigenic (OspA, OspC, p100, VlsE, p39, p58, p18, p41) band revealed on the strip according to their intensity was calculated and interpreted by the test strip analysis software, and scored as negative, positive, or equivocal. Briefly, an IgG/IgM WB test result was considered positive if the sum of the points for each strip had a score  ≥ 8 for IgG (and  ≥ 1 for VlsE) or  ≥ 7 for IgM, and equivocal with a score between 6 and 7 for IgG or a score of 6 for IgM. Both serological assays were performed and interpreted according to the manufacturer’s guidelines [36, 37]. The donor’s serology was considered positive only if the positive or equivocal ELISA result in IgG or IgM was confirmed by a positive WB result. The results were collected as a categorical variable.

#### Statistical analysis

Continuous normally distributed variables were reported as median and interquartile range and categorical variables were presented as frequencies and percentages. Categorical variables were compared using Chi-square tests. For all tests, a level of significance of 0.05 was chosen. Statistical analysis was carried out using Epi Info™ 2000 software.

## Results

### Study group

In total, 1200 human serum samples (representing 0.1% of the total population of the counties investigated [38]), were collected from donors in six counties of Romania (200 samples/county). Blood donors consisted of 794 (66.2%; 95% CI 63.4–68.8) men and 406 (33.8%; 95% CI 31.2–36.6) women. Five hundred sixty-nine (47.4%; 95% CI 44.6–50.3) donors were young adults, 581 (48.4%; 95% CI 45.6–51.2) were middle-aged, and 50 (4.2%; 95% CI 3.2–5.5) were old adults. The median age was 41 years (interquartile range 53–29), with an urban-to-rural ratio of 809:391 (67.4%:32.6%).

According to their job fields (occupation), 143 (11.9%; 95% CI 10.2–13.9) donors engaged in outdoor activities, consequently with potential exposure to tick bites, and 1057 (88.1%; 95% CI 86.1–89.8) engaged in indoor activities, while 659 (54.9%; 95% CI 52.1–57.7) had secondary and 541 (45.1%; 95% CI 42.3–47.9) tertiary levels of education.

#### Serology results

##### ELISA

In total, 191 out of 1200 (15.9%; 95% CI 14.0–18.1) samples had positive ELISA results and 49 (4.1%; 95% CI 3.1–5.4) had equivocal antibody index values. Eighty-nine (7.4%; 95% CI 6.1–9.0) samples presented positive IgG and 122 (10.2%; 95% CI 8.6–12.0) positive IgM antibody index values, and from the 191 positive samples, 20 samples (1.7%; 95% CI 1.1–2.6) were positive for both IgG and IgM antibodies. Seven samples (0.6%; 95% CI 0.3–1.2) had equivocal results for IgG and 49 (4.1%; 95% CI 3.1–5.4) for IgM. Seven (0.6%; 95% CI 0.3–1.2) samples had positive antibody index values for IgG and equivocal values for IgM.

##### WB

Serum samples from 240 (20%; 95% CI 17.8–22.4) donors with positive or equivocal IgG and IgM ELISA index values were further analyzed by WB. Overall, 71 (5.9%; 95% CI 4.7–7.4) samples had positive (4.1%, *n*  = 49; 95% CI 3.1–5.4) and 22 (1.8%; 95% CI 1.2–2.8) had equivocal WB results. Positive WB results represent a mean value of 1.89 cases per million inhabitants for the counties investigated.

**Fig. 1:**
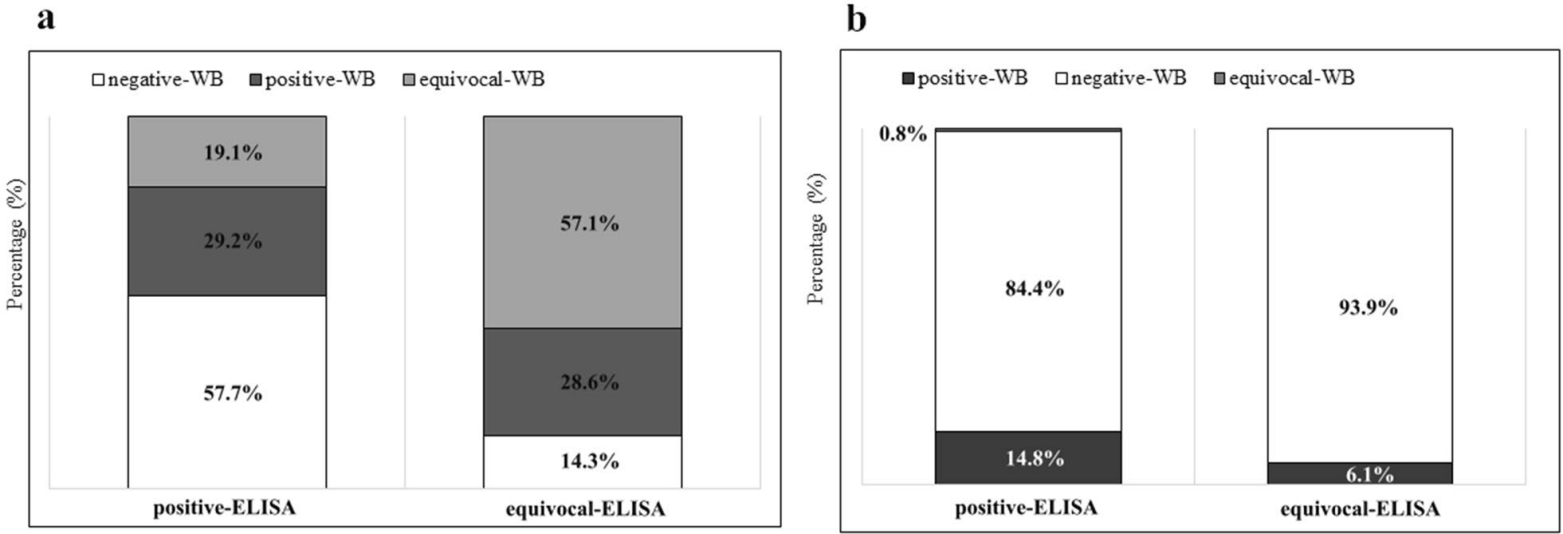
Western blot results for confirmation of ELISA-positive or ELISA-equivocal IgG (**a**) and IgM (**b**) results

In total, 28 (2.3%; 95% CI 1.6–3.4) serum samples tested by WB were positive for *B. burgdorferi* s.l. for IgG and 21 (1.8%; 95% CI 1.2–2.7) for IgM, whereas 21 (1.75%; 95% CI 1.2–2.7) serum samples had equivocal WB results for IgG and one (0.1%; 95% CI 0.0–0.5) for IgM. Twenty-six (29.2%; 95% CI 20.5–39.8) ELISA-positive IgG and two ELISA-equivocal IgG (28.6%; 95% CI 3.7–71.0) serum samples were confirmed by WB. Among the 122 ELISA-positive IgM samples, 18 (14.8%; 95% CI 9.0–22.3) were positive by WB, and one serum sample (0.8%; 95% CI 0.0–4.5) had an equivocal WB result. From the equivocal ELISA IgM results, three (6.1%; 95% CI 1.3–16.9) had positive WB results (Fig. 1).

#### Risk factors

For each age category, seroprevalence was higher in men than in women, and the highest WB positivity rates among immunoblot results for both antibodies were obtained in the old adults (8%), followed by middle-aged (5%) and young adults (2.8%) (Table 1).

**Table 1:** Seroprevalence of *B. burgdorferi* s.l. according to gender, environment, education, and activities, among age groups and in the investigated counties for each age category

Categories	Prevalence % (+/*n*; 95% CI)		
	IgG	IgM	Total
Gender			
F	1.2 (5/406; 0.5–2.9)	1.7 (7/406; 0.8–3.5)	3.0 (12/406; 1.7–5.1)
M	2.9 (23/794; 1.9–4.3)	1.8 (14/794; 1.1–2.9)	4.7 (37/794; 3.4–6.4)
Environment			
U	2.5 (20/809; 1.6–3.8)	2.0 (16/809; 1.2–3.2)	4.5 (36/809; 3.2–6.1)
R	2.1 (8/391: 1.0–4.0)	1.3 (5/391; 0.6–3.0)	3.3 (13/391; 2.0–5.6)
Education			
S	2.3 (15/659; 1.4–3.7)	2.1 (14/659; 1.3–3.5)	4.4 (29/659; 3.1–6.3)
H	2.4 (13/541; 1.4–4.1)	1.3 (7/541; 0.6–2.7)	3.7 (20/541; 2.4–5.6)
Activities			
O	3.5 (5/143; 1.1–8.0)	0.7 (1/143; 0.0–3.8)	4.2 (6/143; 1. 6–8.9)
I	2.2 (23/1057; 1.5–3.2)	1.9 (20/1057; 1.2–2.9)	4.1 (43/1057; 3.0–5.4)
Total	2.3 (28/1200; 1.6–3.4)	1.8 (21/1200; 1.2–2.7)	4.1 (49/1200; 3.1–5.4)
Age groups			
Young			
M	1.3 (5/384; 0.6–3.0)	1.6 (6/384; 0.7–3.4)	2.9 (11/384; 1.6–5.1)
F	1.1 (2/185; 0.1–3.9)	1.6 (3/185; 0.3–4. 7)	2.7 (5/185; 0.9–6.2
Total	1.2 (7/569; 0.60–2.52)	1.6 (9/569; 0.8–3.0)	2.8 (16/569; 1.7–4.5)
Middle-aged			
M	4.3 (16/373; 2.7–6.9)	1.9 (7/373; 0.9–3.8)	6.2 (23/373; 4.1–9.1)
F	1.0 (2/208; 0.1–3.4)	1.9 (4/208; 0.5–4.9)	2.9 (6/208; 1.1–6.2)
Total	3.1 (18/581; 2.0–4.9)	1. 9 (11/581; 1.1–3.4)	5.0 (29/581; 3.5–7.1)
Old			
M	5.411 (2/37; 0.7–18.2)	2.7 (1/37; 0.1–14.2)	8.1 (3/37; 1.7–21.9)
F	7.7 (1/13; 0.2–36.0)	0 (0/13)	7.7 (1/13; 0.2–36.0)
Total	6 (3/50; 1.3–16.6)	2.0 (1/50; 0.1–10.7)	8 (4/50; 2.2–19.2)
Total	2.3 (28/1200; 1.6–3.4)	1.8 (21/1200; 1.2–2.7)	4.1 (49/1200; 3.1–5.4)
Counties			
Alba			
Young	1.1 (1/89; 0.0–6.1)	2.3 (2/89; 0.3–7.9)	3.4 (3/89; 0.7–9.5)
Middle-aged	2.9 (3/104; 0.6–8.2)	2.9 (3/104; 0.6–8.2)	5.8 (6/104; 2.2–12.1)
Old	28.6 (2/7; 3. 7–71.0)	0 (0/7)	28.6 (2/7; 3. 7–71.0)
Total	3 (6/200; 1.1–6.4)	2.5 (5/200; 0.8–5.7)	5.5 (11/200; 2. 8–9.6)
Bistrița-Năsăud			
Young	0 (0/87)	2.3 (2/87; 0.3–8.1)	2.3 (2/87; 0.3–8.1)
Middle-aged	5.6 (6/108; 2.1–11.7)	4.6 (5/108; 1.5–10.5)	10.2 (11/108; 5.2–17.5)
Old	0 (0/5)	0 (0/5)	0 (0/5)
Total	3 (6/200; 1.1–6.4)	3.5 (7/200; 1.4–7.1)	6.5 (13/200; 3.5–10.9)
Cluj			
Young	2.2 (3/139; 0.5–6.2)	1.4 (2/139; 0.2–5.1)	3.6 (5/139; 1.2–8.2)
Middle-aged	0 (0/57)	3.5 (2/6; 0.4–12.1)	3.5 (2/57; 0.4–12.1)
Old	25 (1/4; 0.6–80.6)	0 (0/4)	25 (1/4; 0.6–80.6)
Total	2 (4/200; 0.6–5.0)	2 (4/200; 0.6–5.0)	4 (8/200; 1.7–7.7)
Maramureș			
Young	2 (2/100; 0.2–7.0)	2 (2/100; 0.2–7.0)	4 (4/100; 1.1–9.9)
Middle-aged	3.2 (3/94; 0.7–9.0)	1.1 (1/94; 0.013–5.8)	4.3 (4/94; 1.2–10.5)
Old	0 (0/6)	0 (0/6)	0 (0/6)
Total	2.5 (5/200; 0.8–5.7)	1.5 (3/200; 0.3–4.3)	4 (8/200; 1.7–7.7)
Sălaj			
Young	1.1 (1/92; 0.0–5.9)	0 (0/92)	1.1 (1/92; 0.0–5.9)
Middle	5.0 (5/101; 1.6–11.2)	0 (0/101)	5.0 (5/101; 1.6–11.2)
Old	0 (0/7)	0 (0/7)	0 (0/7)
Total	3 (6/200; 1.1–6.4)	0 (0/200)	3 (6/200; 1.1–6.4)
Satu-Mare			
Young	0 (0/62)	1.1 (1/62; 0.0–8.7)	1.6 (1/62; 0.0–8.7)
Middle	0.9 (1/117; 0.0–4. 7)	0 (0/117)	0.9 (1/117; 0.0–4.7)
Old	0 (0/21)	4.8 (1/21; 0.1–23.8)	4.8 (1.21; 0.1–23.8)
Total	0.5 (1/200; 0.0–2.8)	1 (2/200; 0.1–3.6)	1.5 (3/200; 0.3–4.3)
Total	2.3 (28/1200; 1.6–3.4)	1.8 (21/1200; 1.2–2.7)	4.1 (49/1200; 3.1–5.4)

“+” = number of positive samples; *n* = total number of samples; *F* female; *M* male; *U* urban; *R* rural; *S* secondary education; *H* higher education; *O* outdoor; *I* indoor

The WB produced statistically significant differences regarding the age groups (*χ*^2^  =  6.2978, *df*  =  2; *P*  =  0.0429). Nevertheless, statistically significant results between young and middle-age categories (*χ*^2^  =  3.8788, *df*  =  1; *P*  =  0.0489) were recorded for IgG but not for IgM.

Higher seroprevalence was found in donors from urban environments (4.5%) relative to those from rural environments (3.3%). While 4.4% of the donors with secondary education had positive WB results, the seroprevalence of blood donors with higher education was 3.7%. These differences were not statistically significant.

According to their occupational fields, 4.2% of the donors with outdoor activities and 4.1% with indoor activities had positive WB results (Table 1), and statistically significant differences for IgG (*χ*^2^  =  6.7112, *df*  =  2; *P*  =  0.0349) were recorded.

**Fig. 2:**
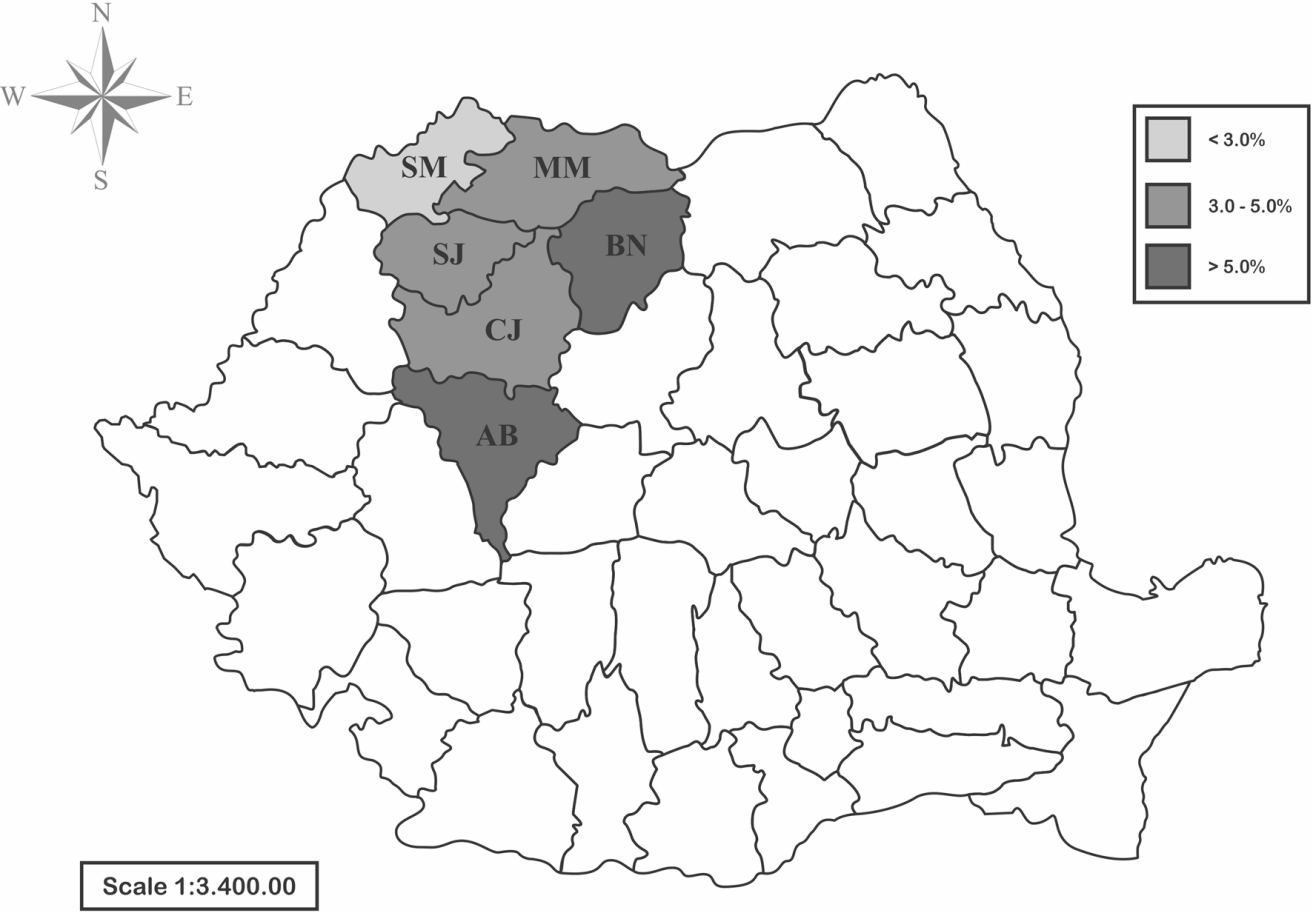
Seroprevalence of *B. burgdorferi* s.l. in blood donors from Romania. *AB* Alba County; *BN* Bistrița-Năsăud County; *CJ* Cluj County; *MM* Maramureș County; *SJ* Sălaj County; *SM* Satu-Mare County

The seroprevalence of *B. burgdorferi* s.l. among donors did not differ significantly by county, and varied between 1.5% (Satu-Mare) and 6.5% (Bistrița-Năsăud) (Fig. 2; Table 1). However, statistically significant results for IgG seroprevalence by age group were registered in Alba County (*χ*^2^  =  17.2993, *df * =  4; *P*  =  0.0017) and for the young age group within the six counties (*χ*^2^  =  19.613, *df*  = 10; *P*  =  0.0331).

### Discussion

The true incidence of LB around the world is thought to be higher than the number of reported cases. While LB is frequently misdiagnosed, overdiagnosis also often occurs in healthcare systems [39]. In certain European countries, heterogeneity is found among surveillance systems [8]. In Europe, over 85,000 cases of LB are estimated to occur each year [40]; however, incidence rate and seroprevalence vary substantially between the countries and even among different regions within one country.

Seroprevalence results for IgG antibodies against *B. burgdorferi* s.l. among different populations have been reported in several European countries (1.1% in Belgium, 2.7% in Germany, 3.2% in France and Sweden, 4.9% in Italy and Finland) [44–49]. However, comparisons of the results are limited by the use of different kits with different sensitivity and specificity, and also differences between batches of the same kit [41]. Another limitation in seroprevalence studies is the difference in the study groups used (e.g., forestry workers, farmers, soldiers), as diverse and representative sampling for the whole population can be expensive. Except for Lyme neuroborreliosis, there is no harmonized surveillance for LB in Europe, with large differences from country to country regarding the type of reporting (mandatory vs. non-mandatory), type of surveillance (active vs. passive), case definition, and confirmatory laboratory tests. This lack of harmonized data is the principal reason for the limited ability for data comparison.

Over the past 10 years, reports of tick-borne pathogens, including LB-causing *Borrelia* species, have increased significantly in Romania. Briciu et al. reported a 9.1% seroprevalence of *B. burgdorferi* s.l. antibodies in humans after a tick bite, and in another study they evaluated the clinical and serological outcome of two groups of patients 1 year after tick bite [13, 42]. In other studies, the prevalence of *B. burgdorferi* s.l. in questing *I. ricinus* varied between 0.6 and 40% in different localities [14–16, 43], with the highest prevalence reported in forested but arid areas in the southeastern part of Romania. An infection prevalence of 3.3% was reported for *B. burgdorferi* s.l. in *I. ricinus* collected from patients in Sibiu [44], with rates varying between 11.1 and 12.4% in Cluj County, one of the counties investigated in the present study [13, 18].

In Romania, the only similar study with a large dataset (in total *n* = 2666) has evaluated the seroprevalence of *B. burgdorferi* IgG antibodies in sera collected from forestry workers and healthy blood donors [12]. Hristea et al. studied 1598 healthy blood donors from 13 districts from Romania, from which three counties (Alba, Bistrița-Năsăud, Maramureș) were also included in the present study. In the previous report, the seroprevalence of IgG antibodies was 4.3% among the blood donors and 9.3% among the forestry workers. In the present study, 2.3% of the blood donors had positive IgG and 1.8% had positive IgM antibody responses to *B. burgdorferi*, as determined by the two-test approach. Although equivocal results of WB IgG (1.8%) and IgM (0.1%) were recorded, a follow-up evaluation of patients, as recommended in this case, was not possible to perform. We also evaluated the IgM seroprevalence against *Borrelia burgdorferi* in our analyses, as our previous studies on erythema migrans showed persistence of IgM antibodies at 1-year follow-up in 35% (ELISA) and 27% (WB) of cases, while an important percentage of the patients presented IgG serological results on both ELISA (17%) and WB (12%) [35]. Nonetheless, a high frequency of false-positive IgM results is also known from previous studies [45].

Compared to our results, Hristea et al. [12] reported slightly higher seroprevalence in two of the investigated counties (8% vs. 6.5% in Bistrița-Năsăud, 8.7% vs. 4% in Maramureș), but not in Alba County (1.4% vs. 5.5%). The differences in seroprevalence may be related to the different diagnostic assays used in the two studies. Although Hristea et al. used the same two-tiered strategy, indirect hemagglutination assay was performed as a first-step assay, and a different commercially available WB kit was used. WB kits seem to be highly variable and clearly differ among manufacturers. Therefore, different two-step assay combinations and different commercially available kits may influence test results between laboratories, even in the same sample groups [46, 47].

Some studies have used healthy blood donors as a control group compared to patients from groups at risk (soldiers, forestry workers, farmers) to determine the seroprevalence of *B. burgdorferi* s.l. IgG and IgM antibodies. However, routine serological screening for *Borrelia* spp. in healthy people in the absence of clinical data is not recommended, for several reasons [48]: (1) specific IgG antibodies can persist for years after the initial infection or active LB [32]; (2) in follow-up studies, patients with known LB and antibiotic treatment can still have IgG response several years after disease onset [32, 49]; (3) it is known that IgM antibodies may be detected in higher levels in the initial phase of the infection, but residual levels of IgM may be present as well in the late stage of LB [9, 32–35]; (4) a positive IgM WB result may also indicate a false-positive result or a cross-reaction and not an infection, thus leading to overdiagnosis and unnecessary antibiotic therapy in cases of incorrect interpretation [39, 50]; and (5) seroreactivity may decline over a long period of time. Moreover, a positive WB result does not distinguish an active infection from a past infection. Nonetheless, seroprevalence studies within risk groups or risk areas are still a key indicator and may provide a useful alternative to surveillance data for LB [8].

Habitat heterogeneity, climatic differences in the region favorable for tick vectors, and the prevalence of *Borrelia* spp. infection in ticks are also instrumental in determining local disease risk [1]. All the investigated counties in the present study have a typical landscape favorable for *I. ricinus*, with hills and mountain regions. Seroprevalence was lower in donors from the two northern counties (Satu-Mare and Maramureș) than in donors from the northwestern (Bistrița-Năsăud, Cluj, Sălaj) or central counties (Alba).

The smaller number of blood donors from rural areas relative to the urban population in the present study may be the result of the limited access to blood donor centers among people from rural areas. It is known that rural populations in general live in closer proximity to tick habitats, and thus the incidence of tick bites and LB in rural areas is high [51, 52]. In the present study, the seroprevalence in donors residing in urban areas was higher compared to rural areas. In Romania, foci of *Borrelia*-infected *I. ricinus* ticks and small mammals as reservoir hosts have been identified in metropolitan areas, urban parks, and recreational hotspots as well [17, 43, 53]. These findings support the higher seroprevalence results found in the present study for donors from urban areas who engage in recreational activities outdoors. Although the seroprevalence rate was higher in urban areas, a higher seroprevalence was found in blood donors who perform their professional activities outdoors.

Analysis of LB seroprevalence has shown variability between genders. Although the difference in prevalence by gender was not statistically significant in the present study, a higher seroprevalence in men was observed. This may be related to the higher exposure to tick bites during their outdoor employment and professional and leisure activities. The association of seroprevalence with different age groups has also been reported in several studies, showing that LB has a bimodal age distribution; thus the most affected age groups are the youngest (children) and the older citizens [54–56]. The sampling fraction regarding age category was not uniform among the age categories in the present study, and the total number of old donors was the lowest (4.2%); thus statistically significant differences were found between the age groups. However, the old age group had the highest seroprevalence for both antibodies. In addition, several studies have shown that an increase in seroprevalence with age reflects the population’s cumulative exposure to *B. burgdorferi* s.l. [12, 55, 56]. While studies in France, Finland, and Norway reported that the incidence of LB was higher in woman [51, 57–59], in a cross-sectional study conducted using historical serum samples in Finland [11], the highest seroprevalence was observed in men and in persons aged  ≥ 50 years. Similar results have been found in several European countries [12, 54, 56, 60], with younger and older men the most affected groups [56].

The limitations of the present study include the small size of the study group compared to the population of Romania, the regional area included, and the lack of information on self-reported tick bite or diagnosis of LB in the medical history that could allow for a better interpretation of the serological results. Seroprevalence studies using blood donor study groups have limitations due to the exclusion and inclusion criteria as well as the social structure of the donors [61]. Thus, further studies with the inclusion of risk groups of patients from our region are needed. Because a positive IgG or IgM response may persist years after *B. burgdorferi* s.l. infection, these results cannot be interpreted clinically. A follow-up study with clinical evaluation of the subjective health complaints of patients with seropositivity identified by the two-tiered test could provide valuable information on LB. Also, a population-based sampling survey might provide a more representative nationwide sample; however, it may be time-intensive and expensive.

### Conclusions

This study represents an update of the LB seroprevalence for the northwestern and central parts of Romania, showing that seroprevalence has not increased in the last 20 years among blood donors. The results are likely to have a public health impact, with the aim of giving an overall picture of exposure within the country and allowing for comparison with similar studies from the EU. However, further studies are needed with the inclusion of risk groups of patients and collection of tick bite history and clinical data.

## Data Availability

Not applicable.

## Abbreviations

LB: Lyme borreliosis
ELISA: Enzyme-linked immunosorbent assay
WB: Western blot
CI: Confidence interval
